# Does the inspiratory muscle warm-up have an acute effect on wrestling recovery performance?

**DOI:** 10.1371/journal.pone.0316821

**Published:** 2025-02-24

**Authors:** Erkan Demirkan, Mehmet Ismail Tosun, Abdurrahim Kaplan, Irem Eker Arici, Halit Harmanci, Michael Favre, Damian George Cosmin, Veysi Aslan

**Affiliations:** 1 Department of Coaching Education, Faculty of Sport Sciences, Hitit University, Corum, Türkiye; 2 Department of Physical Education and Sports, Faculty of Sport Sciences, Hitit University, Corum, Türkiye; 3 Department of Pulmonary Diseases, Faculty of Medicine, Hitit University, Corum, Türkiye; 4 Departmen of Coaching Education, Faculty of Sport Sciences, Kutahya Dumlupinar University, Kütahya, Türkiye; 5 Intercollegiate Athletics, University of Michigan, Ann Arbor, Michigan, United States; 6 Department of Physical Education and Sports, Faculty pf Physical Education and Sport, Ovidius University of Constanta, Constanța, Romania; 7 Department of Coaching Education, Graduate School of Health Sciences, Ege University, Türkiye; University of Tennessee Health Science Center College of Graduate Health Sciences, UNITED STATES OF AMERICA

## Abstract

This study aims to investigate the acute effects of inspiratory muscle warm-up (IMW) in young wrestlers. Wrestling is a high-intensity sport that demands anaerobic metabolism, with rapid recovery and endurance playing crucial roles in subsequent performance. Inspiratory muscle warm-up specifically targets the inspiratory muscles, reducing fatigue during exercise and helping to sustain performance. Our study compares three different warm-up protocols (traditional wrestling warm-up, wrestling warm-up (WW_IW_) +  IMW, and wrestling warm-up +  placebo (WW_PL_)) to analyse changes in inspiratory muscle strength and select respiratory function parameters. The study was conducted with 14 male wrestlers aged 15-16. Participants were subjected to the three different warm-up protocols, followed by simulated wrestling bouts. Results showed that the WW_IW_ protocol increased maximal inspiratory pressure by 17.3% compared to the traditional and placebo warm-ups. Additionally, the WW_IW_ protocol delayed fatigue and improved recovery rates among the wrestlers. Specifically, WW_IW_ enabled a faster return to normal heart rate post-competition, accelerating the recovery process. These findings suggest that WW_IW_ can be effectively used in high-intensity sports like wrestling to enhance recovery between matches and improve overall performance. Further studies with larger sample sizes and in different sports are recommended to validate these results.

## Introduction

Wrestling is a sport that includes an exceptional dynamic in the activity between competitors with a prominent change of tempo and rhythm during combat. A wrestler mainly performs in the zones of maximal and submaximal load during both competition and training [1]. Therefore, wrestling is a complex sport that requires a high level of aerobic capacity to ensure rapid recovery and increase the anaerobic threshold level [2,3,4,5]. From the aspect of energy, the sport of wrestling predominantly utilizes anaerobic glycolytic pathways during the competitive bout. Previous literature [2,6] has shown that there was a unique high level of metabolic stress on the body, whereby wrestlers during combat reach the blood lactate level of about 10–20 mmol·L- 1, and commonly exhibit heart rates (HR) nearing max. According to the present wrestling rules for the under (U) 15 and U17 age group categories, the duration of a bout is two periods of 2 minutes each with a 30-second break. In comparison, for U20, U23 and seniors, the duration of a bout is two periods of 3 minutes each with a 30-second break [7]. The recovery, which is supported by aerobic metabolism, is an important process to replenish the energy stores during and after high-intensity exercise [8]. The rate and quality of recovery are extremely important for the high-performance athlete, as optimal recovery may provide numerous benefits such as the performance of subsequent high intensity efforts [8,9,10]. The use of heart rate variability is known as a crucial tool to monitor the status of training and recovery after a loading process [11].

The warm-up, including passive, general, or specific warm-up, before the event or training is a necessary activity to prevent sports-related injuries and to enhance physical performance. However, some studies [8,12] reported that a specific IMW protocol was more effective to enhance inspiratory muscle strength than a whole-body warm-up protocol. Inspiratory muscle warm-up optimizes oxygen use by reducing fatigue of respiratory muscles and contributes to rapid recovery by preventing the accumulation of metabolic wastes [13,14]. Warming-up the inspiratory muscles before exercise allows these muscles to fatigue less during exercise, and thus allows high-intensity efforts to be sustained for longer [15]. Additionally, IMW reduces the use of anaerobic glycolytic pathways by increasing oxygen uptake, which is critical for sports such as wrestling [16,17]. This process delays the accumulation of lactic acid, thereby postponing the decline in performance and accelerating recovery [18]. Therefore, IMW exercises, in addition to a whole-body warm-up, have been used as important tools to enhance exercise and sports performance [8]. The literature has also reported that IMW enhances inspiratory muscle (IM) function [10,19,20], improves exercise tolerance [10], increases running distance performance [20], decreases lactate concentration during exercise [19], and reduces dyspnea [10,19]. Additionally, the specific IMW protocol leads to greater protection against decreasing muscle oxygen saturation during both the submaximal cycling exercise and the subsequent intermittent high-intensity sprint exercise [17]. The reason for conducting the research with young wrestlers is that the physiological development of this group continues, and the long-term performance effects of interventions made during this period are more critical [21].

It may be advantageous for wrestlers to develop quick recovery strategies between and after competitions at an early age. Faster recovery is believed to lead to starting the competition in a more energized state and experiencing less fatigue later. Therefore, revealing the effects of IMW on young wrestlers will contribute to the determination of strategies suitable for the developmental processes. The aim of this research is to examine the acute effects of warm-up for respiratory muscles on recovery, respiratory muscle strength, along with some respiratory function parameters in young wrestlers. Within the scope of the research, it was hypothesized that IMW would increase the recovery speed of young wrestlers after the bout, contribute to the resistance of the respiratory muscles against fatigue, and create positive changes in some respiratory function parameters.

## Methods

### Study design

**Fig 1:**
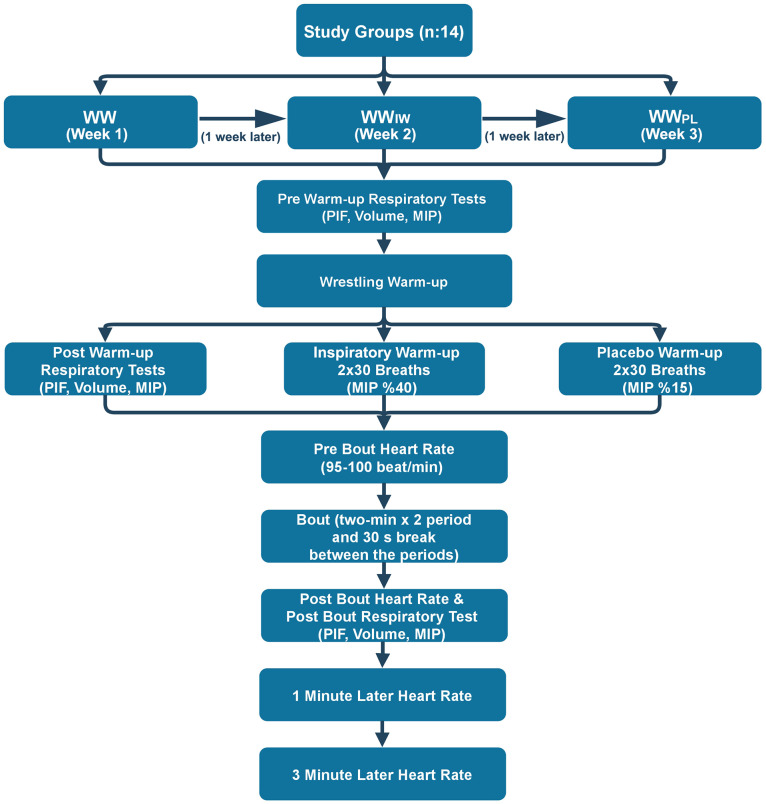
Flow chart of study design.

The study used a single-blind, repeated-measures crossover study design. All warm-up protocols and tests were completed in one day. All study participants were given a comprehensive introduction to the tests to be administered as part of the research 12 and 6 days prior to the start of the study, in addition trial measurements were conducted. Participants were briefed that they would perform one wrestling warm-up protocol and two identical inspiratory muscle warm-up protocols added to the wrestling warm-up. Participants were informed that they would undergo three distinct warm-up protocols. One protocol consisted solely of a standard warm-up, while the other two included an additional inspiratory muscle warm-up alongside the standard routine. The two inspiratory muscle warm-up protocols were identical in terms of their application method. Participants were further informed that the purpose of performing two respiratory muscle warm-ups was to assess the reliability and repeatability of this protocol when combined with the standard warm-up. However, of these two inspiratory muscle warm-up protocols, one was a main inspiratory muscle warm-up, and the other was a placebo. In our study, three different warm-up protocols (Wrestling Warm-up, Wrestling Warm-up +  Inspiratory Muscle Warm-up, Wrestling Warm-up +  Placebo Warm-up) were administered on the same day and time of the week (Monday, 2–3:00 pm) with a one-week interval between warm-up protocol. A simulated wrestling bout (two-min x 2 period, with a 30 s break between the periods) was performed after the warm-up protocol and measurements, according to the United World Wrestling rules, between wrestlers of the same weight class. The simulated wrestling bout lasted until the end of two-minute duration, regardless of any pin that might occur, ensuring consistency in match length. Additionally, subjects competed exclusively against the same opponent within their weight class throughout all protocol applications, with no changes in opponents. In future studies, alternative wrestling tests that allow for more controlled load management could be considered. Participants were encouraged to deliver peak performance during the competitions. Maximal inspiratory pressure (MIP) tests were conducted to assess inspiratory muscle strength, peak inspiratory flow (PIF), and inspiratory volume both immediately before and after the warm-up protocols. Additionally, participants’ heart rate data were recorded before the competition, immediately after, and 1 minute and 3 minutes post-competition (Fig. 1). Before the warm-up protocols, participants rested for 24 hours and fasted for 4 hours. Recruitment of participants began on July 3, 2023, and ended on July 17, 2023. Written informed consent forms, approved by the Ethics Committee for Research at Hitit University, were obtained from all participants, as well as written consent forms from the parents or guardians of the participants. Ethical approval for the study was obtained from the Hitit University Research Ethics Committee prior to the commencement of the research (Decision Number: 2023-07, Decision Date:05.06.2023). In addition, the Declaration of Helsinki has been adhered to throughout the study.

### Participant

Prior to beginning the study, the sample size was calculated with the G Power 3.1.9.7 (Dusseldorf, Germany) program which concluded that 12 participants were sufficient to represent the group with a power of 95% (β=0.95), an effect size (f) of 0.534 and an error rate of α=0.05. To eliminate the loss of potential participants, 14 young male wrestlers with at least 4 years of wrestling history (age 15.21 ±  0.42, height 170.5 ±  5.16, body mass 67.1 ± 10.09, body mass index 22.96 ±  2.87) participated in the study. All participants train at the same sports club, under the guidance of the same coach, five days a week. The inclusion criteria for the study were as follows: being between 14–16 years of age, having participated in regular wrestling training for at least 4 years, being male, having no health problems that would prevent participation in the study, no history of chest diseases, being a non-smoker, and not having sustained any sports injuries in the past 12 months.

### Study procedure

Participants who volunteered to join the study and met the inclusion criteria completed the necessary paperwork (Informed Consent Form – Informed Parental/Guardian Consent Form). After receiving approval from the principal investigator, the warm-up protocols and tests to be conducted within the scope of the study were initiated. In our study, three different warm-up protocols were applied in the following order: wrestling warm-up, wrestling warm-up plus inspiratory warm-up, and wrestling warm-up plus placebo warm-up. Each protocol was separated by a one-week break and their impacts on inspiratory muscle strength, peak inspiratory flow, inspiratory volume, and recovery were evaluated. The warm-up protocols were as follows:

**Wrestling warm-up (WW).** It was designed as a traditional specific wrestling warm-up that contained jogging (10-minute), followed by dynamic stretching exercises (10 minute) for the upper and lower body extremities. After the heart rate reached 95–100 beats/min with the traditional wrestling specific warm-up, then the wrestling bout was performed.

**Wrestling warm-up plus inspiratory warm-up.** It was designed so the WW_IW_ protocol was coupled together with the traditional specific wrestling warm-up. All participants first performed the wrestling warm-up, then immediately carried out the respiratory warm-up by performing two sets of 30 breaths with inspiratory pressure-threshold equivalent 40% maximum inspiratory mouth pressure using the POWERbreathe® respiratory muscle device (POWERbreathe International Ltd., U.K.) [22]. After the heart rate reached 95–100 beats/min with the wrestling warm-up plus respiratory specific warm-up, then the wrestling bout was performed.

**Wrestling warm-up plus placebo warm-up.** The same method was followed as with the WW_IW_, but the POWERbreathe® respiratory muscle device was set by inspiratory pressure- threshold equivalent to 15% without notice for the participants [22]. After the heart rate reached 95–100 beats/min with the wrestling warm-up plus placebo warm up specific warm-up, then the wrestling bout was performed.

In both the placebo and Wrestling Warm-up Plus Respiratory protocols, participants were not informed about the intensity of respiratory muscle warming during the warm-up. Participants were blinded to this issue. Participants were invited to the WW_PL_ protocol to assess the repeatability and reliability of the previous WW_IW_ protocol. Although participants believed they were following the same protocol, the training intensities differed.

### Test protocols

#### Measurement of inspiratory muscle strength.

Maximal inspiratory pressure which reveals the force generating capacity of inspiratory muscles during the Muller maneuver, is a valid method used to measure respiratory muscle strength [12,23]. This measurement was determined with a portable device (Micro Medical-Carefusion Micro RPM, United Kingdom) [24]. The measurement score can be monitored instantly from the device. Participants were asked to hold the measuring instrument with both hands and close their lips tightly around the flanged mouthpiece during the measurement. The participants were asked to exhale as deeply as possible (residual volume) and then perform maximum inspiration for more than one second for the test [23,25]. A nose clip was applied to prevent air escaping from the nose, and the test was performed while the participants were standing [26]. Participants completed three MIP trials at each timepoint, and the highest value was recorded and used for statistical analysis.

### Peak inspiratory flow and inspiration volume test

Peak inspiratory flow evaluates the ability of inspiratory muscles to contract rapidly and overcome resistance inherent in the respiratory system [27]. Inspiratory volume is the volume and amount of air reaching the lungs with each inspiration [28]. A portable device (POWERbreathe K5, United Kingdom) was used for both tests [29]. The test was performed with the participant standing with his nose closed with a clothespin and was asked to exhale as deeply as possible (residual volume) and then perform maximum inspiration for more than one second for the test. Peak inspiratory flow and inspiration volume were both taken simultaneously in the single breath test mode of the device [30,31,32].

### Heart rate monitoring

Heart rate was measured just before and immediately after the performance bouts using a commercially available pulse oximeter (PO80, Beuer Germany) device that was placed tightly on the forefinger without moving and recorded the data [33,34].

### Statistical analysis

The results are presented as mean and standard deviation, and 95% confidence intervals. The normality of distribution and homogeneity of variances of the main variables were confirmed using a Shapiro-Wilk normality test and Levene’s test, respectively. To evaluate the differences between the three warm-up protocols, one-way ANOVAs and Tukey HSD post hoc test were used to assess differences between the three different warm-up protocols. Two-way repeated measures analysis of variance (ANOVA) was applied to compare the repeated measurements in a group and used a Bonferroni test as post hoc test. Values of p < 0.05 were considered statistically significant. All statistical analyses were conducted using the SPSS 22 (IBM Corp., Armonk, NY, USA).

## Results

**Fig 2:**
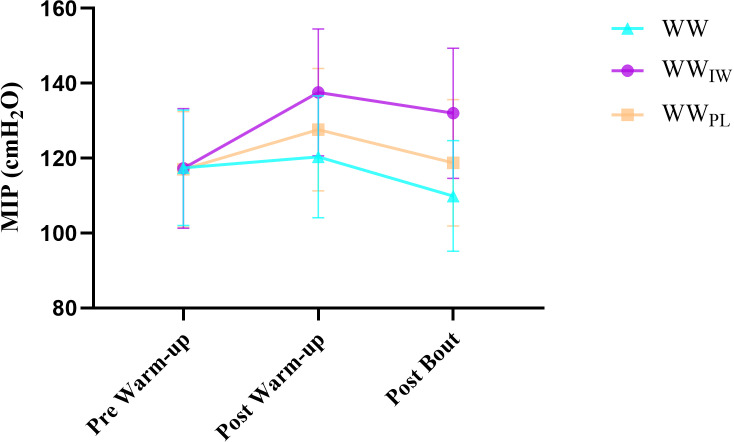
Maximal inspiratory pressure values of the participants according to the warm-up protocols.

**Fig 3:**
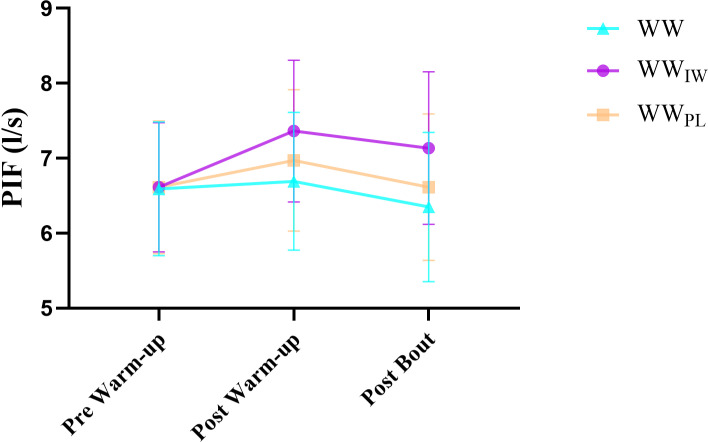
Peak inspiratory flow values of the participants according to the warm-up protocols.

**Fig 4:**
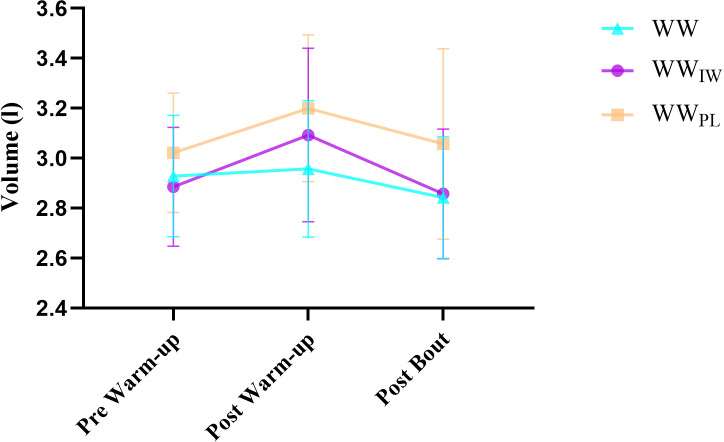
Inspiratory Volume values of the participants according to the warm-up protocols.

Table 1 presents both the results of the warm-up protocols (WW, WW_IW_, WW_PL_) and repeated measures (pre-warm up, post warm up and post bout). According to the protocols, the differences were not statistically significant between all protocols in the MIP that performed the pre-warm up (p = 0.993). In both the post-warm up and post-bout, there was a significant difference between the WW and WW_IW_ in MIP (p = 0.02, p = 0.002) (Fig. 2). In the assessment of the PIF and the respiratory volume for each of the WW, WW_IW_, WW_PL_ protocols in the inter-groups (pre-post and after the bout), there were no significant differences among all protocols for both the PIF (p = 0.993, p = 0.144, p = 0.071 respectively) and the respiratory volume (p = 0.322, p = 0.124, p = 0.123 respectively). The results of repeated measures showed there were statistically significant differences in MIP for both WW and WW_IW._ While in the WW_PL_ group there was a significant difference between post-warm up and post bout (p = 0.05). A significant difference was found in PIF of both post-warm-up and post bout according to the pre-warm-up in WW_IW_ (p = 0.05) (Fig 3). In the WW group, a statistically significant difference was found in post bout of the PIF, according to both pre-warm up and post warm-up (p = 0.05). Post warm-up PIF was significantly different from pre-warm-up and post bout in WW_PL_ (p = 0.05). In the inspiratory reserve volume, the WW group had a statistically significant lower post bout measurement than the pre and post warm-up (p = 0.034). However, in WW_IW_, there was found to be a significant difference in post warm-up in comparison to pre and post bout (p = 0.034) (Fig 4). Details are presented in Table 1.

**Table 1:** Participants’ Maximal Inspiratory Pressure (MIP), Peak Inspiratory Flow (PIF) and Volume test values.

Value	Protocol	Pre Warm-up	% Dif	Post Warm-up	% Dif	Post Bout	p
**MIP** **(cmH**_**2**_ **O)**	**WW**	^1^117.3 ± 15.4	2.3	^2^120^b^ ± 16.3	-8.9	^3^109.3^a^ ± 15.2	0.005
	**WW**_**IW**_	^1^117.2 ± 15.9	17.3	^2^137.5^a^ ± 16.9	-4	^3^132^b^ ± 17.3	0.005
	**WW**_**PL**_	^1^117.9 ± 15.3	7.8	^2^127.1^b^ ± 14.3	-7.6	^3^117.4^b^ ± 14.7	0.005
**p**		0.993		0.020		0.002	
**PIF** **(l/s)**	**WW**	^1^6.5 ± 0.89	3.1	^1^6.7 ± 0.90	-6.0	^2^6.3 ± 1.02	0.010
	**WW**_**IW**_	^1^6.6 ± 0.86	10.6	^2^7.3 ± 0.94	-2.7	^2^7.1 ± 1.01	0.005
	**WW**_**PL**_	^1^6.5 ± 0.79	4.6	^2^6.8 ± 0.91	-5.9	^1^6.4 ± 0.88	0.010
**p**		0.993		0.144		0.071	
**Volume** **(Liters)**	**WW**	^1^2.9 ± 0.24	0	^1^2.9 ± 0.27	-3.4	^2^2.8 ± 0.24	0.034
	**WW**_**IW**_	^1^2.8 ± 0.23	7.1	^2^3 ± 0.34	-6.7	^1^2.8 ± 0.25	0.034
	**WW**_**PL**_	3.0 ± 0.23	6.7	3.2 ± 0.29	-6.3	3.0 ± 0.38	0.123
**p**		0.322		0.124		0.123	

**p < 0.05; Dif:** Difference; **WW**: Wrestling Warm-up; **WW**_**IW**_: Wrestling Warm-up Plus Respiratory Muscle Warm-up; **WW**_**PL**:_ Wrestling Warm-up Plus Placebo Respiratory Muscle Warm-up; **MIP:** Maximal Inspiratory Pressure; **PIF:** Peak Inspiratory Flow; **Volume:** The inspiratory reserve volume is the amount of air a person can inhale forcefully after normal tidal volume inspiration (litres).

**Fig 5:**
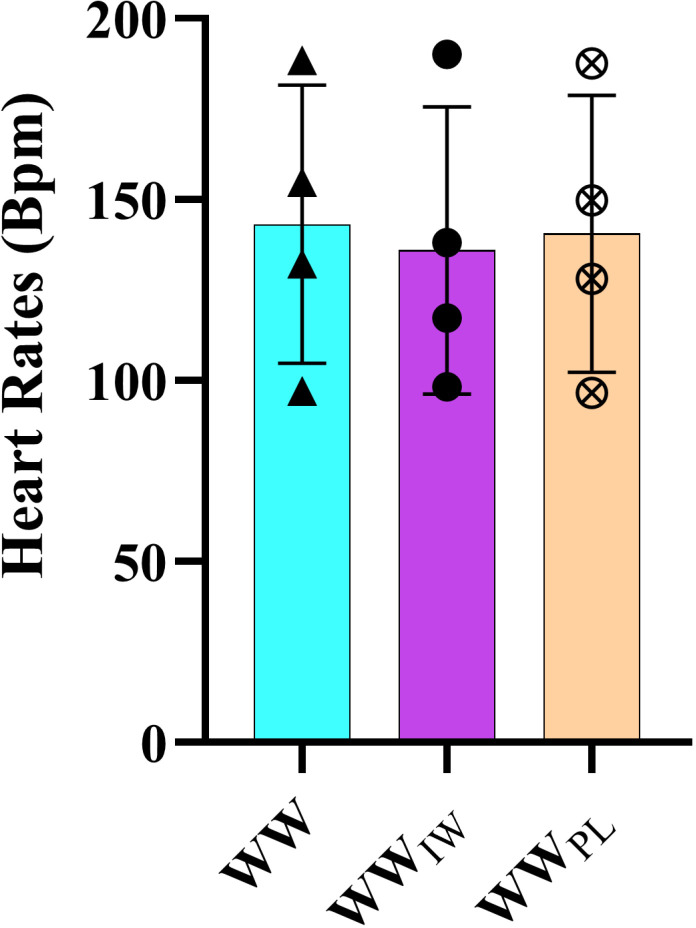
Heart rates values of the participants according to the warm-up protocols.

Table 2 presents the values that compared the heart rates pre and post bout in first and third minutes. For the heart rate values in pre and post bout, there was no significant difference among the protocols (p = 0.795, p = 0.625). In addition, it was seen that there was a statistically significant difference in the first and third minutes after the bout in all groups (p = 0.001). While comparing within the protocols, there were found to be statistically significant differences among all the periods (pre-post bout and after the first and third minutes) (p = 0.001) (Fig 5). Details are presented in Table 2.

**Table 2:** The pulse values of the wrestlers based on the warm-up protocols.

Protocol	Pre Bout	% Dif	Post Bout	1 min. later	% Dif	3 min later	% Dif.	p
**WW**	^1^97 ± 6.31	94.2	^2^188.4 ± 8.32	^3^154.7^c^ ± 3.9	-17.8	^4^132.2^b^ ± 9.15	-29.8	0.001
**WW**_**IW**_	^1^98.2 ± 7.84	93.5	^2^190.1 ± 6.70	^3^138^a^ ± 5.2	-27.4	^4^117.2^a^ ± 5.22	-38.3	0.001
**WW**_**PL**_	^1^96.6 ± 4.32	88.2	^2^187.5 ± 6.08	^3^149.7 ^b^ ± 3	-20.1	^4^128^a-b^ ± 6.48	-31.7	0.001
**p**	0.795		0.625	0.000		0.000		

p < 0.05; Dif: difference; min: minute; **WW**: Wrestling Warm-up; **WW**_**IW**_: Wrestling Warm-up Plus Respiratory Muscle Warm-up; **WW**_**PL**:_ Wrestling Warm-up Plus Placebo Respiratory Muscle Warm-up.

## Discussion

To the best of our knowledge, this is the first study to compare the effects on respiratory muscle pressure and recovery heart rates with three different protocols consisting of WW, WW with respiratory warm-up (WW_IW_) and WW with placebo (WW_PL_) during the simulated wrestling bout. During the assessment of the effects between the pre and post warm-up on MIP, WW and WW_PL_ protocols resulted in an increase of 2.3% and 7.8% respectively, while the most powerful increase occurred in WW_IW_ protocol with 17.3% (Table 1). However, in the values of the respiratory pressure after the bout, WW and WW_PL_ protocols showed a return to 8.9%, and 7.6% respectively, while WW_IW_ decreased 4% (Table 1). The decrease of the respiratory pressure may be due to the WW_IW_. Based on these findings, it could be thought that the inspiratory muscle warm-up firstly led to the powerful increase in the MIP, and then resulted in an important delaying in respiratory muscle fatigue compared to other protocols in the post bout. This result was seen clearly in the repeated measures of WW_IW_ (pre-warm-up:117.2 ± 15.9 post warm-up:137.5 ± 16.9, and post bout: 132 ± 17.3). In previous literature studies, Volianitis et al. [12] studied the effects of respiratory warm-up on rowing performance, finding that the respiratory warm-up led to a 7% enhancement in MIP in comparison to the baseline values of the inspiratory muscle strength. In addition, they reported that respiratory warm-up combined with the specific rowing warm-up significantly reduced fatigue to 4.2% compared with submaximal rowing warm-up (10.2%) and a specific rowing warm-up (11.1%). In a similar study conducted by Tong et al. [10] they investigated the effects of IMW together with a whole-body warm-up protocol on the maximum dynamic inspiratory muscle (IM) function. This study’s [10] findings indicated that IMW improved in the pressure parameters to include the individual’s MIP at zero flow (P0), maximal inspiratory muscle power (WImax), optimal pressure (Popt) relative to P0 (Popt/P0), and maximal rate of pressure development. Lomax et al. (20) reported that IMW increased MIP by the rate of 11%, along with increasing running distance by 5-7%. They also stated that the highest increase occurred when the IMW and inspiratory muscle training were performed together. In another study, Wilson et al. [22] stated that the inspiratory muscle exercise combined with a standard swimming warm-up significantly improved swimming performance in elite swimmers. Özdal et al. [14] stated that respiratory warm-up exercise significantly improved anaerobic power (peak power) and peaked faster. Barnes & Ludge [35] reported that the IMW when compared with a standard warm-up, showed a small positive effect (~21 seconds, 2.8%) on 3200m running performance. Manchado-Gobatto et al. [18] stated that the pre-activation of the inspiratory muscles at 40% of the maximal inspiratory pressure, improved the running power and enhanced recovery.

Furthermore, in our investigation of the flow parameters resulting from the warm-up protocols, WW_IW_ protocol improved the peak inspiratory flow (PIF) 10.6% between the pre-warm-up and post warm-up, after that it provided a 2.7% decline in PIF in the post bout. However, the WW and WW_PL_ protocols caused a higher decline (6.0%, 5.9% respectively) in PIF in the post bout according to the WW_IW_ (Table 1). This lower rate, which occurred post bout in the peak inspiratory flow, suggests that WW_IW_ could be caused by more resistance against fatigue than the WW and WW_PL_ protocols (Table 1). These findings indicate a plausible explanation is that the mechanisms responsible for these acute improvements that occurred at the end of the bout may be associated with the post-activation potentiation effect in inspiratory muscle.

In this context, some contrary studies were reported. Merola et al. [36] conducted a study in judo athletes that showed that the respiratory muscle warm-up had no effect on special judo fitness test performance. Richard & Billaut [37] researched the effects of IMW in elite speed skaters performing 3000m on-ice time trials. The results of IMW compared with the placebo intervention indicated no impact on skating time and tissue oxygen saturation index. The lack of statistically significant effects in both studies may be attributed to the inclusion of elite athletes as participants, along with differences in the methods applied within the test protocols. The respiratory muscle strength and overall performance of elite-level athletes are often already at their peak. Additionally, sports such as speed skating create high intramuscular pressure in athletes’ muscles, which may limit blood flow, thus limiting the potential benefits of IMW. Cheng et al. [17] reported that IMW showed no significant improvement on ventilatory and metabolic responses during the submaximal cycling exercise and intermittent high-intensity sprint exercise in female soccer players. However, they demonstrated that the specific IMW exercise (40% MIP, 2 sets of 30 breaths) protected against the decrease in muscle oxygen saturation in the submaximal cycling exercise and intermittent high intensity exercise. The finding that IMW did not have a significant effect on high-intensity intermittent sprint performance in this study may be related to the higher oxidative capacity of respiratory muscles in female athletes, which results in less fatigue of these muscles during exercise, thereby limiting the contribution of IMW to performance. Additionally, the increased workload on the diaphragm during fixed-position exercises like cycling may negatively affect performance by restricting blood flow to the locomotor muscles through the respiratory muscle metaboreflex. A similar study conducted by Ohya et al. [38] found that specific IMW exercise had the effect of improving IM function, but it had no effect in high intensity intermittent sprint cycling exercise performance in untrained males. In the study, subjects were defined as individuals who performed recreational exercise (such as soccer, cycling, swimming, and running) three times per week, but were not professionally trained. These individuals did not have a history of high-intensity training, which may be a crucial factor in evaluating the findings of the study and the effects of respiratory muscle warm-up. Since the capacity and demand of respiratory muscles are higher in trained athletes, warm-up may have a greater effect on these muscles. Therefore, the subjects’ training levels may have caused these differences. Faghy & Brown [39] showed that IMW enhanced maximal inspiration pressure, but it did not lead to an ergogenic effect in load carriage time-trial performance when performed alone or together with a whole-body active warm-up. The impairment of respiratory mechanics due to load carriage may have constrained the performance of the respiratory muscles, and consequently, the potential benefits of the warm-up. In this context, the study suggests that IMW did not yield the anticipated performance improvements in specific load-bearing tasks, and that such interventions may exhibit variable effects depending on the type of exercise, its duration, and the nature of the load.

In a review study conducted by Cirino et al. [13], they reported that 88% of the studies that related to IMW had positive effects on inspiratory parameters, and 45% of those were related to performance. A probable reason for the contrast of the findings could be the study design. Our study design was performed to the conditions of the simulated wrestling bouts, but the other studies [17,20,36,38] previously mentioned, were conducted with a specific performance test. This situation could be the reason for the discrepancy. When the respiratory muscle findings in our study were assessed together with the heart rate values, the study findings related to the heart rates between post bout and one minute later showed that WW_IW_ protocol provided a rapid decline in heart rate variability of 27.4% compared to the WW and WW_PL_ (17.8%, 20.1% respectively) (Table 2). In addition, the WW_IW_ protocol also caused a rapid decline in the heart rate variability (38.3%) between the 1-minute post-bout and after the third minute, in comparison to the WW and WW_PL_ protocols (29.8%, 31.7% respectively) (Table 2). Based on these findings, the delaying of fatigue (4%) in respiratory muscle (Table 1) and the rapid decline in heart rate variabilities (after one minute: 27.4% and three minute later:38%) that occurred in the WW_IW_ protocol may be related to the respiratory sinus arrhythmia. The heart rate recovery findings in our study may be supported by the inspiration volume values in Table 1. It showed that both protocols (WW_IW_ and WW_PL_) provided the pre-warm-up baseline inspiration values after the post bout, but WW protocol resulted in a lower inspiration volume than the pre-warm-up. The physiological explanation of the findings in our study and the effects of the WW_IW_ protocol may arise not only from the positive effects of warm-up exercises for respiratory muscles on respiratory muscle strength, but also from their contribution to autonomic nervous system modulation. Inspiratory muscle warm-up increases parasympathetic nervous system activity, thereby suppressing sympathetic activity. This effect leads to a more rapid decline in heart rate and an improvement in heart rate variability, thereby accelerating the post-exercise recovery process [15]. The effect of respiration on vagal tone can be enhanced by diaphragmatic breathing techniques, which help to reduce heart rate rapidly and regulate blood pressure by increasing baroreflex sensitivity [40,41]. Diaphragmatic breathing is known to support the autonomic nervous system’s regulatory mechanisms and suppress sympathetic tone by reducing stress responses [42,43].

Further search of the literature found that Reis et al. [44] reported that the respiratory sinus arrhythmia was due to the heart rate following an oscillatory pattern synchronized with the respiratory cycle. In addition, this phenomenon, based on these findings, may suggest that parasympathetic activity after the WW_IW_ increased at rest when compared to the WW and WW_PL_. In a study conducted by Rodrigues et al. [45], they reported that inspiratory muscle training provided a positive effect on cardiac autonomic control through a vagal predominance at rest during spontaneous breathing in older women, but detraining reversed it to the baseline values. In another study, it was reported by Tanriverdi et al. [46] that a single session of diaphragmatic breathing exercise significantly improved heart rate and heart rate variability after inspiratory muscle training at 60% of MIP. Pal & Velkumary [47] stated that slow breathing exercise training for a minimum of three months improved autonomic nervous system activity by increasing parasympathetic activity and decreasing sympathetic activity. Cheng et al. [17] reported that low intensity (10% maximal inspiratory mouth pressure) inspiratory resistive loading treatment improved metabolic acidosis conditions after high-intensity interval sprints. They suggested that inspiratory resistive loading could be used as a training tool in the cool-down routines after sprint interval training. Marostegan et al. [8] reported that 40% of the MIP improved tissue saturation index of the biceps brachii muscle during the recovery phase, which could indicate higher availability of oxygen for lactate clearance. According to our findings, along with those within the literature, we suggest that the WW_IW_ protocol may be used for delaying fatigue in respiratory muscle and eliciting faster recovery with the rapid decline in heart rate.

## Conclusion

The mechanisms responsible for these acute improvements that occurred at the end of the bout, may be associated with the post-activation potentiation effect in inspiratory muscle. Based on this study’s findings, the inspiratory muscle warm-up exercise may be used, especially in combat sports (wrestling, judo, taekwondo etc.), before the bout, and between rounds to provide fast recovery. However, to clarify these findings, it suggests that studies need to be conducted in future with more participants in different sports branches.

### Limitation

The study consisted of young male wrestlers who were the range of 15 to 16 age years and had at least four years training experiences. The study was conducted in a simulated wrestling bout condition. Inspiratory Muscle Warm-up protocol was performed with the POWER breathe® Classic Medium Resistance respiratory muscle training device during the study. It was used Beuer po 80 device to determine the heart rate values in pre- and post-bout, and after first and third minute.

## Supporting information

S1 Data on the wrestlers in the WW Protocol. (XLSX)

S2 Data on the wrestlers in the WW(_IW_) Protocol. (XLSX)

S3 Data on the wrestlers in the WW(_PL_) Protocol. (XLSX)
